# Molecular surveillance reveals a potential hotspot of tick-borne disease in Yakeshi City, Inner Mongolia

**DOI:** 10.1186/s12866-023-03110-6

**Published:** 2023-11-20

**Authors:** Junhua Tian, Jing Liu, Hongqing Zhao, Xiaomin Chen, Xueqin Geng, Miao Lu, Kun Li

**Affiliations:** 1 Wuhan Center for Disease Control and Prevention, Wuhan City, Hubei Province 430024 China; 2 National Key Laboratory of Intelligent Tracking and Forecasting for Infectious Diseases, National Institute for Communicable Disease Control and Prevention, Chinese Center for Disease Control and Prevention, Changping District, Beijing City, 102206 China; 3 Caidian Center for Disease Control and Prevention, Wuhan City, Hubei Province 430100 China; 4 National Institute for Communicable Disease Control and Prevention, Chinese Center for Disease Control and Prevention, Changping Liuzi 5, Beijing, 102206 China

**Keywords:** Ticks, *Rickettsia*, Anaplasmataceae, *Borrelia*, *Candidatus* Anaplasma mongolica

## Abstract

A molecular surveillance of tick-borne diseases was performed in Hulunbuir City, Inner Mongolia. A total of 149 ticks including three species (*Ixodes persulcatus*, *Haemaphysalis concinna*, and *Dermacentor silvarum*) were collected. As many as 11 tick-borne bacterial pathogens were identified in them. Some of them have high positive rates. For example, *Candidatus* Rickettsia tarasevichiae was detected with a high prevalence of 72.48%, while *Candidatus* Lariskella sp. was detected in 31.54% of ticks. For both *Rickettsia raoultii* and *Anaplasma phagocytophilum*, two distinct genotypes were identified based on their phylogenetic trees based on 16S rRNA, *gltA*, and *groEL* sequences. Remarkable genetic diversity was also observed for 16S and *flaB* genes of *Borreliella garinii*, an agent of Lyme disease. *Rickettsia heilongjiangensis* causing Far-Eastern spotted fever (2.68%, 4/149), *Ehrlichia muris* causing human ehrlichiosis (4.70%, 7/149), *Borrelia miyamotoi* causing relapsing fever (2.01%, 3/149), and *Borreliella afzelii* causing Lyme disease (2.01%, 3/149) were also detected. Additionally, a previously uncharacterized *Anaplasma* species closely related to *Anaplasma ovis* was identified. Herein we name it “*Candidatus* Anaplasma mongolica”. Based on these results, we propose that Yakeshi City might be a potential hotspot of tick-borne diseases.

**Supplementary Information:**

The online version contains supplementary material available at 10.1186/s12866-023-03110-6.

## Introduction

Emerging tick-borne diseases pose a threat to public health worldwide. To date, at least 124 tick species have been recorded in mainland China, including 113 hard tick species in seven genera and 11 soft tick species in two genera [1]. Accordingly, these tick species have been reported to carry at least 103 tick-borne agents, most of which were detected in the past two decades [1]. The order Rickettsiales and the genus *Borrelia* are the largest groups of tick-borne bacterial pathogens. Rickettsiales mainly composed of genera *Rickettsia*, *Anaplasma*, and *Ehrlichia*. It includes a large number of important tick-borne pathogens, such as *Rickettsia rickettsii*, *Rickettsia raoultii*, *Ehrlichia chaffeensis*, *Anaplasma phagocytophilum*, *Anaplasma capra*, and *Candidatus* Neoehrlichia mikurensis. Until 2021, at least 21 *Rickettsia*, nine *Anaplasma*, eight *Ehrlichia*, and one *Candidatus* Neoehrlichia species (or variants) were detected in ticks from China. Of those, *A. phagocytophilum*, *E. chaffeensis*, and *R. raoultii* parasitize the largest number of tick species (reported in 22, 16, and 15 tick species, respectively) [1]. Human cases infected by these pathogens were also frequently reported. From 2009 to 2010, 46 human cases infected with *A. phagocytophilum* were laboratory-confirmed in Beijing, Hebei, and Shandong provinces, North China [2]. In addition to the well-known pathogens, some newly identified members of Rickettsiales are increasingly reported to infect humans. As recently as in 2022, *Candidatus* Midichloria mitochondrii, previously known as an endosymbiont of hard ticks, was detected in blood samples of 34.1% of humans with a tick bite history, suggesting that the genetic diversity and human pathogenicity of various Rickettsiales bacteria still warrant further explorations [3]. The genus *Borrelia* is closely related to lots of human diseases. Since 2014, the genus *Borrelia* was divided into two genera: the genus *Borrelia* containing the members of the relapsing fever *Borrelia* (*Borrelia miyamotoi*, *Borrelia theileri*, *Borrelia persica*, etc.), and the genus *Borreliella* containing the Lyme disease *Borrelia* (*Borrelia burgdorferi* sensu lato, *Borrelia afzelii*, etc.) [4]. Of those, Lyme borreliosis caused by the Lyme disease group is considered the most common tick-borne disease in the Northern Hemisphere. Approximately 476,000 human cases were reported in the USA each year [5]. In China, at least nine genospecies of *B. burgdorferi* have been documented, most of which were detected in ticks [6].

The Inner Mongolia Autonomous Region located in the north of China is rich in various wildlife and has a diverse ecosystem. Due to the vast territory (1, 183, 000 km^2^) and unique geographical/ecological features, this is one of the several regions that harbor the most abundant tick species in China [1]. Accordingly, it is one of the major epidemic areas of tick-borne infectious diseases in China including tick-borne encephalitis, anaplasmosis, rickettsiosis, Lyme disease, and babesiosis [7]. Although lots of studies have been performed in ticks from Inner Mongolia, Rickettsiales bacteria and other tick-borne pathogens in many tick species and many cities in this area are still not extensively characterized. In this study, we collected three tick species from Hulunbuir City in northeast Inner Mongolia and studied the potential human pathogens in them.

## Methods

### Sample collection and DNA extraction

**Fig. 1:**
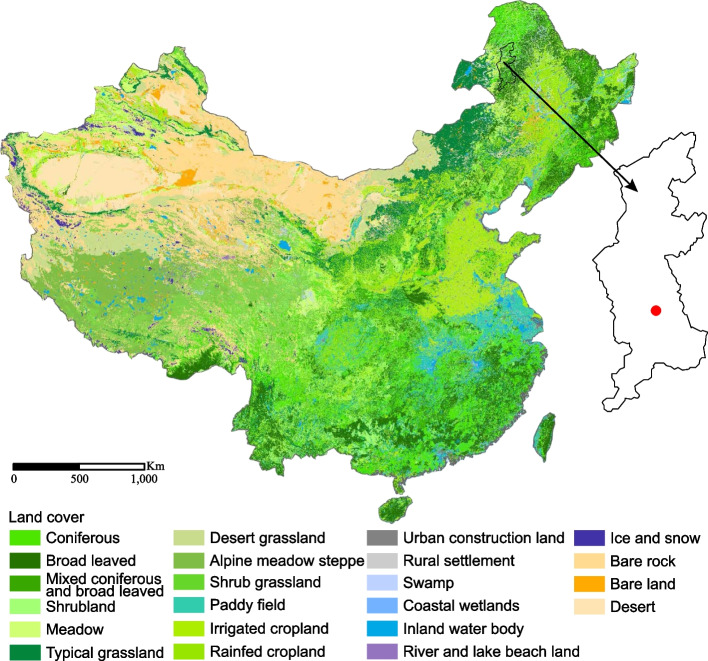
A map showing the location where the ticks were collected

In 2018, ticks were collected in the Ilekd Village, Wunuer Town, Yakeshi County-Level City of Hulunbuir City, Inner Mongolia (Fig. 1). This location is located in forest areas of the Greater Khingan Range, with an average altitude of approximately 850 m. The ticks were carefully removed from the body surface of free-ranging goats and cattle using tweezers and then transported alive to Wuhan Center for Disease Control and Prevention. Based on the taxonomic characters described in previous literature, the tick species were initially determined by morphological observation of their palp, scutum, anal groove, and shape of basis capitulum. All the ticks were observed by a stereoscopic microscope (Olympus SZX 16, Olympus Corporation, Tokyo, Japan). For further confirmation, ticks were randomly selected from each species, and the *COI* (Cytochrome oxidase I) sequences were PCR amplified and sequenced (primers shown in reference [8]) after DNA extraction.

Before DNA extraction, each tick was washed twice using phosphate buffer saline (PBS) to exclude possible environmental contamination, and then individually ground into homogenate manually in a mortar with 100 μL PBS. Total DNA was extracted from the homogenates using a Mollusc DNA Extraction Kit (Omega Bio-Tek, USA) following the instructions. To test the quality of DNA extraction, the DNA concentration was measured by spectrophotometric analysis (Thermo Scientific Nanodrop, Delaware, USA).

### Molecular detection and identification of the pathogens

The DNA samples were screened for the presence of *Rickettsia*, Anaplasmataceae (*Anaplasma* spp., *Ehrlichia* spp., etc.), and *Borrelia* bacteria. Primers used were shown in previous reports [9–11], generating PCR products with a size of approximately 900 bp, 450 bp, and 400 bp, respectively. The *Rickettsia* and Anaplasmataceae were detected targeting the 16S rRNA gene, while *Borrelia* was screened targeting the *flaB* (flagellin B) gene. For precise identification, the *gltA* and *groEL* (60 kDa chaperonin) sequences were amplified from the *Rickettsia*, *Ehrlichia*, and *Anaplasma* strains detected in this study using primers as shown [9, 10]. Furthermore, 16S rRNA sequences (approximately 1200 bp) were amplified from the *Borrelia* strains [11], and *gltA* sequences (approximately 400 bp) were amplified from the *Candidatus* Lariskella strains [9].

### Genetic and phylogenetic analysis

To calculate the nucleotide similarities and determine their species, all the nucleotide sequences recovered in this study were manually aligned with reference sequences in the GenBank Database by BLASTn. The representative reference sequences were downloaded from the database. After alignment by Mega 6.0, the nucleotide sequences were phylogenetically analyzed based on the maximum likelihood (ML) method by PhyML3.0 [12]. All the phylogenetic trees were visualized and edited using FigTree v1.4.3.

## Results

### Sample collection and species identification

**Fig. 2:**
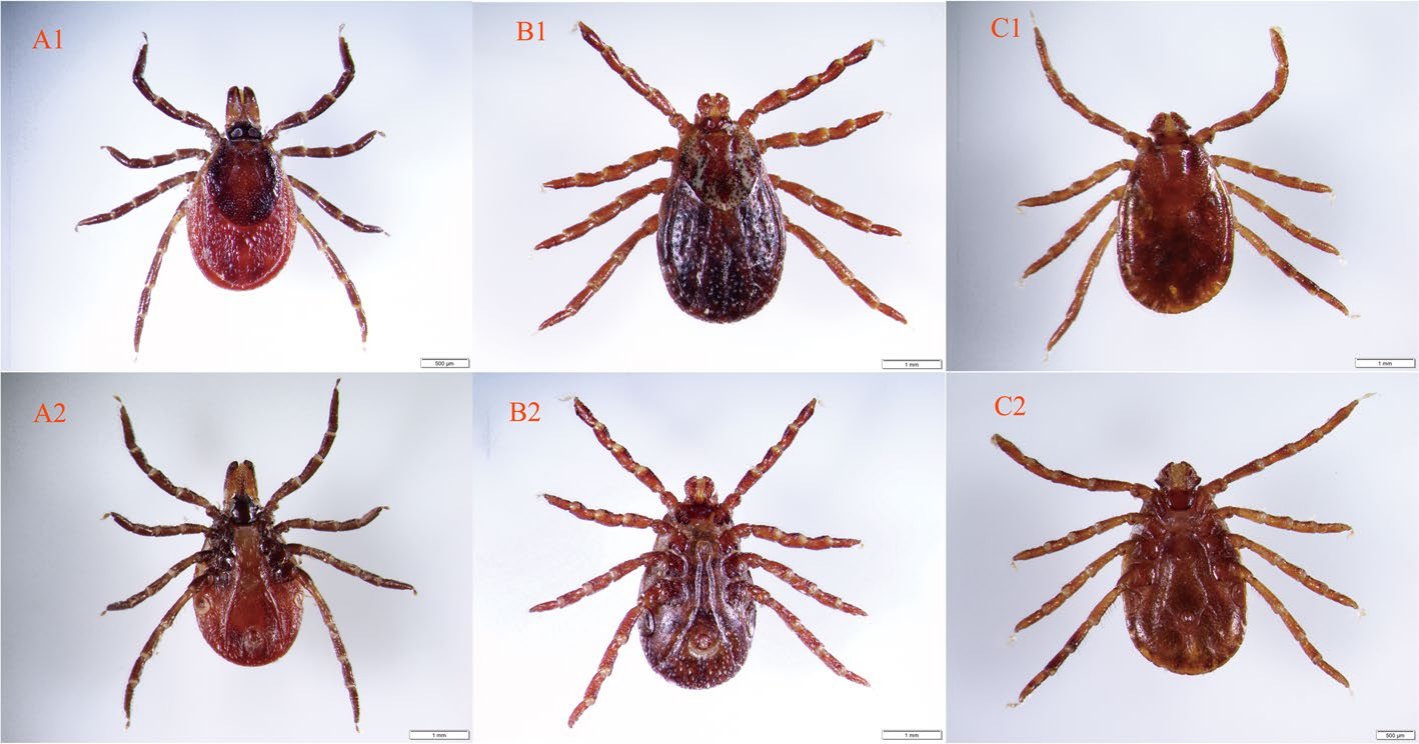
The photographs of ticks under a stereoscopic microscope. **A** *Ixodes persulcatus*. **B** *Dermacentor silvarum.* **C** *Haemaphysalis concinna*

In May 2018, a total of 149 ticks were collected from the body surface of ten cattle and eight goats in the Yakeshi County-Level City of Hulunbuir City, Inner Mongolia (49.17°N, 120.40°E). Based on morphological observation, three species were identified: 99 *Ixodes persulcatus*, 24 *Haemaphysalis concinna*, and 26 *Dermacentor silvarum* (Fig. 2). For further confirmation, the *COI* sequences of all three tick species have nucleotide similarities higher than 99% with reference sequences.

### Detection and analysis of the *Rickettsia* spp.

Based on the PCR results and sequencing of 16S rRNA sequences, a total of three *Rickettsia* species including four genotypes were identified. *Candidatus* Rickettsia tarasevichiae was detected in all *I. persulcatus* (99/99, 100%), 7 of 24 *H. concinna* (29.17%), and 2 of 26 *D. silvarum* (7.69%) (Table 1). All the 16S sequences are 100% identical to *Ca.* Rickettsia tarasevichiae isolate Dog-145, *Ca*. Rickettsia tarasevichiae isolate Bayan-68, and *Ca*. Rickettsia tarasevichiae isolate Mulan-11, which were all detected in the Heilongjiang Province of Northeast China. Similarly, the *gltA* (1007 bp) sequences show highest 99.90% nucleotide similarity to these strains, and the *groEL* (1060 bp) sequences have 99.65–99.88% (coverage 76–80%) to previously uploaded sequences (Accession numbers: ON863711, OP722688, MN450404, and MN450402).

**Table 1:** Prevalence of *Rickettsia* spp., *Anaplasma* spp., *Borreliella* spp., *Borrelia miyamotoi*, *Ehrlichia muris*, and *Candidatus* Lariskella sp. in different tick species from Hulunbuir City of Inner Mongolia

	*Ixodes persulcatus*	*Haemaphysalis concinna*	*Dermacentor silvarum*	Total
*Candidatus* Rickettsia tarasevichiae	99/99 (100%)^a^	7/24 (29.17%)	2/26 (7.69%)	108/149 (72.48%)
*Rickettsia raoultii* type I	0/99 (0.00%)	0/24 (0.00%)	13/26 (50.00%)	13/149 (8.72%)
*Rickettsia raoultii* type II	0/99 (0.00%)	0/24 (0.00%)	7/26 (26.92%)	7/149 (4.70%)
*Rickettsia heilongjiangensis*	0/99 (0.00%)	4/24 (16.67%)	0/26 (0.00%)	4/149 (2.68%)
*Anaplasma phagocytophilum* type I	1/99 (1.01%)	0/24 (0.00%)	0/26 (0.00%)	1/149 (0.67%)
*Anaplasma phagocytophilum* type II	3/99 (3.03%)	0/24 (0.00%)	0/26 (0.00%)	3/149 (2.01%)
*Anaplasma bovis*	0/99 (0.00%)	1/24 (4.17%)	0/26 (0.00%)	1/149 (0.67%)
*Candidatus* Anaplasma mongolica	1/99 (1.01%)	0/24 (0.00%)	0/26 (0.00%)	1/149 (0.67%)
*Ehrlichia muris*	7/99 (7.07%)	0/24 (0.00%)	0/26 (0.00%)	7/149 (4.70%)
*Candidatus* Lariskella sp.	47/99 (47.47%)	0/24 (0.00%)	0/26 (0.00%)	47/149 (31.54%)
*Borrelia miyamotoi*	3/99 (3.03%)	0/24 (0.00%)	0/26 (0.00%)	3/149 (2.01%)
*Borreliella afzelii*	3/99 (3.03%)	0/24 (0.00%)	0/26 (0.00%)	3/149 (2.01%)
*Borreliella garinii*	8/99 (8.08%)	0/24 (0.00%)	0/26 (0.00%)	8/149 (5.37%)

^a^ Positive samples/total samples

**Fig. 3:**
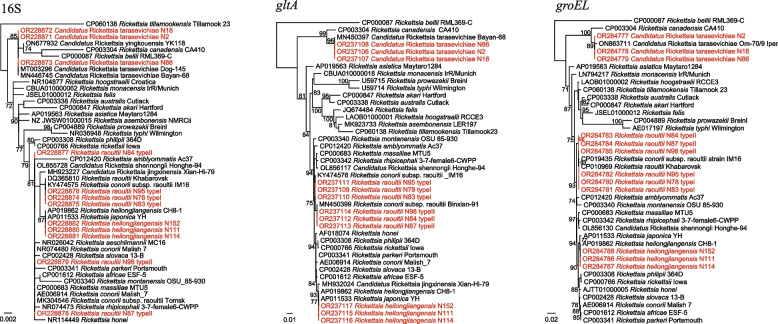
Phylogenetic trees based on the nucleotide sequences of 16S rRNA, *gltA*, and *groEL* genes of *Rickettsia* spp.

Two genotypes of *R. raoultii* were detected only in *D. silvarum*, with positive rates of 50.00% (13/26) and 26.92% (7/26), respectively. All three genes of type I (strains N78, N83, and N95) were 100% identical to *Rickettsia conorii* subsp. raoultii strain IM16. In contrast, the 16S sequences of type II were 100% identical to *Rickettsia conorii* subsp. raoultii isolate Tomsk, *R. conorii* strain Malish_7, and *R. massiliae* MTU5, while their *gltA* sequences were 100% to that of *R. raoultii* isolate Binxian-91. As shown in Fig. 3, these strains clearly divided into two distinct clades.

Four *H. concinna* ticks were tested positive for *R. heilongjiangensis*. All their 16S rRNA and *gltA* sequences show 100% and 99.90% identities to both *R. heilongjiangensis* CH8-1 and *R. japonica* strain YH_M. For the *groEL* sequences, they are 100% identical to those of *R. heilongjiangensis* CH8-1 and *R. heilongjiangensis* HCN-13, but only 99.53% to *R. japonica* strains. These results confirmed that these strains should be classified as *R. heilongjiangensis*.

### Detection and analysis of the *Anaplasma* spp.

**Fig. 4:**
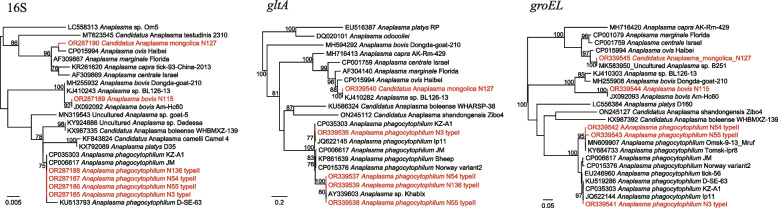
Phylogenetic trees based on the nucleotide sequences of 16S rRNA, *gltA*, and *groEL* genes of *Anaplasma* spp.

Based on analysis of the 16S sequences, a total of three *Anaplasma* species were detected: *A. phagocytophilum*, *A. bovis*, and an unclassified *Anaplasma* sp., showing 100%, 100%, and 99.87% to *A. phagocytophilum* str. JM, *A. bovis* clone Am-Hc60, and *A. centrale* isolate LP10, respectively. Notably, based on *gltA* and *groEL* sequences, the *A. phagocytophilum* strains divided into two types in the phylogenetic trees: *A. phagocytophilum* N3 represent type I while strains N54, N55, and N136 belong to type II (Fig. 4). The *gltA* sequences of type II are 100% identical to *Anaplasma* sp. KhabIx detected in the Russian Far East, and only 82.73–88.11% to *A. phagocytophilum* strains. Similarly, the *groEL* sequence of type I (strain N3) was 100% identical to *A. phagocytophilum* isolate Ip11, but sequences of type II have 98.59–100% similarities to *A. phagocytophilum* strains identified in Tomsk and Omsk in Russia. These results showed the genetic diversity of *A. phagocytophilum* in this area. Out of our expectation, the *gltA* sequence of *Anaplasma* sp. N127 was 100% identical to *Anaplasma* sp. BL126-13, but its *groEL* sequence has a long genetic distance to that of *Anaplasma* sp. BL126-13 (Fig. 4). In contrast, it was 100% identical to the *groEL* sequence of *Anaplasma* sp. clone B251. The 23S sequence of *Anaplasma* sp. N127 was also obtained, showing highest 96.79% identity to *A. ovis* str. Haibei and 94.81% to *A. marginale* str. Florida (Fig. S1). We propose it as a novel species, namely “*Candidatus* Anaplasma mongolica”.

### Detection and analysis of the *Ehrlichia* sp. and *Lariskella* sp.

**Fig. 5:**
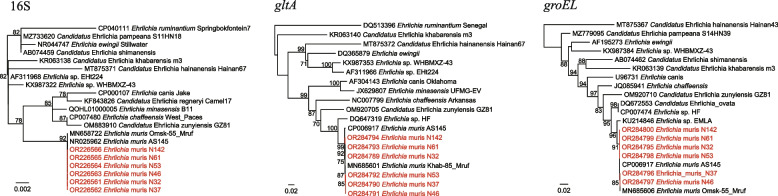
Phylogenetic trees based on the nucleotide sequences of 16S rRNA, *gltA*, and *groEL* genes of *Ehrlichia muris*

Seven *I. persulcatus* ticks were tested positive for *Ehrlichia*, and all of them were identified as *Ehrlichia muris*. In addition to the 16S rRNA (456 bp) sequences which show 100% to *E. muris* strains, the *gltA* (986 bp), and *groEL* (1121 bp) sequences were also successfully obtained. The *gltA* sequences have highest 99.47–99.80% identities to *E. muris* strains in rodents (isolate Khab-85_Mruf, AS145) and *I. persulcatus* ticks (isolate Omsk-563_Ip) from Japan and Russia. The *groEL* sequences are also highly homologous to *E. muris* strains from Japan and Russia, with nucleotide identities of 99.29–100% (Fig. 5). To date, there is only one *E. muris* sequence in the GenBank Database from mainland China.

**Fig. 6:**
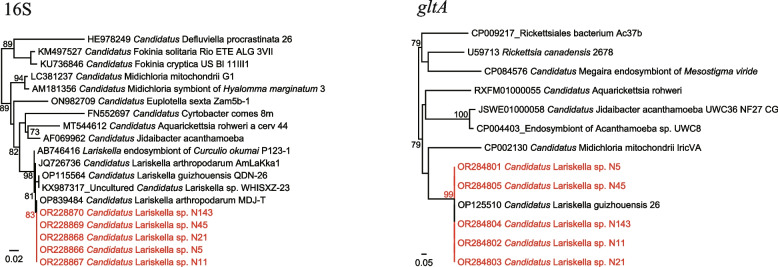
Phylogenetic trees based on the nucleotide sequences of 16S rRNA and *gltA* genes of *Candidatus* Lariskella sp.

Unexpectedly, *Candidatus* Lariskella sp. belonging to the family *Candidatus* Midichloriaceae, order Rickettsiales, was detected in as many as 47 of the 99 *Ixodes persulcatus* ticks (47.47%) using the primers screening Anaplasmataceae. All the 16S rRNA sequences are 100% identical to each other and have 98.83–100% identity to *Candidatus* Lariskella arthropodarum, 98.82–99.06% to *Candidatus* Lariskella guizhouensis we previously reported. Interestingly, their *gltA* sequences (400 bp) are only highly homologous to *Ca.* Lariskella guizhouensis, with similarities of 99.50–99.73% (Fig. 6).

### Detection and analysis of the *Borrelia* sp. and *Borreliella* spp.

**Fig. 7:**
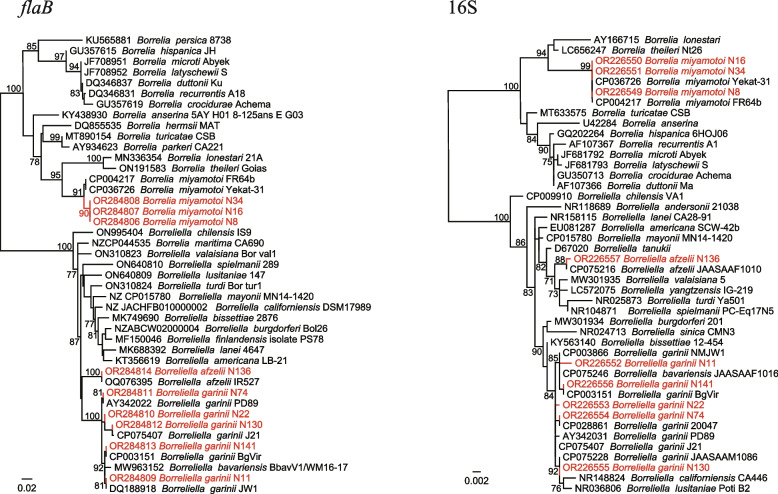
Phylogenetic trees based on the nucleotide sequences of *flaB* and 16S rRNA genes of *Borrelia miyamotoi* and *Borreliella* spp.

One *Borrelia* sp. (*Borrelia miyamotoi*) and two *Borrelialla* spp. (*Borreliella afzelii* and *Borreliella garinii*) were detected. As shown in Table 1, all of them were detected in *I. persulcatus*, with positive rates of 3.03% (3/99), 3.03% (3/99), and 8.08% (8/99), respectively. The *flaB* and 16S sequences of *B. miyamotoi* strains were all closely related to *B. miyamotoi* strain Yekat-31 from Russia, with nucleotide similarities of 98.72% and 100%, respectively. Notably, phylogenetic analysis indicated that both the *flaB* and 16S gene sequences of *B. garinii* showed remarkable genetic diversity (shown in Fig. 7).

### Co-infections of tick-borne pathogens

Because all *I. persulcatus* ticks were positive for *Ca.* Rickettsia tarasevichiae, ticks infected with *A. phagocytophilum* (four strains), *Ca.* Anaplasma mongolica (one strain), *E. muris* (seven strains), *Candidatus* Lariskella sp. (47 strains), *Borrelia* (three strains), and *Borrelialla* (11 strains) strains detected in *I. persulcatus* were all co-infected with *Ca.* Rickettsia tarasevichiae. Of those, one tick infected with *B. miyamotoi* is co-infected with *Ca.* Rickettsia tarasevichiae and *Candidatus* Lariskella sp., while one infected with *B. aafzelii* is co-infected with *Ca.* Rickettsia tarasevichiae and *A. phagocytophilum* (type II). Furthermore, in the nine ticks infected with *B. garinii*, two are co-infected with *Ca.* Rickettsia tarasevichiae and *Candidatus* Lariskella sp., and one is co-infected with *Ca.* Rickettsia tarasevichiae and *Ca.* Anaplasma mongolica. No co-infection was observed in *H. concinna* and *D. silvarum*.

## Discussion

Inner Mongolia has been recognized as an endemic region of tick-borne diseases. To date, numerous studies have been carried out on tick-borne pathogens circulating in this area [7, 13–18]. However, most of these studies only focused on one or two pathogens, and exhaustive investigations on their genetic characteristics are still very few. In this study, we detected and identified as many as 11 tick-borne bacterial pathogens in three tick species from Hulunbuir City, Inner Mongolia. The abundance of tick-borne pathogens, as well as the high positive rates and remarkable genetic diversity of some pathogens, clearly suggest the risk to public health in this area. Furthermore, apparent host specificity was observed for most of these pathogens. For example, seven bacterial species were only detected in *I. persulcatus*. It was consistent with the previous report that *I. persulcatus* is only second to *H. longicornis* as the carrier of tick-borne agents [1].

All three *Rickettsia* species in this study are well-recognized human pathogens. *Candidatus* Rickettsia tarasevichiae is first detected in *I. persulcatus* ticks from Russia in 2003 [19]. After then, tens of human cases infected by this *Rickettsia* were reported in Heilongjiang (northeast China) and Henan (eastern central China) provinces, with the major symptoms of fever, malaise, and anorexia [20, 21]. As previously reported, the tick hosts of *Ca.* Rickettsia tarasevichiae are *I. persulcatus*, *I. sinensis*, *H. longicornis*, *H. concinna*., and *D. silvarum* [1]. It is of note that in this study, the positive rate in different tick hosts varies dramatically, suggesting that the distribution and density of *I. persulcatus* may be the major risk factors for *Ca.* Rickettsia tarasevichiae infections. *Rickettsia heilongjiangensis* and *R. raoultii* are both spotted fever group *Rickettsia.* As the etiologic agent of Far-Eastern spotted fever, *R. heilongjiangensis* has been detected in multiple tick species. However, it is most frequently reported in *H. concinna* ticks, which is consistent with our results [22]. Interestingly, we found two genotypes of *R. raoultii* in *D. silvarum*. One is closely related to *Rickettsia conorii subsp. raoultii* strain IM16. In contrast, the 16S, *gltA*, and *groEL* genes of the other type all show different positions in the phylogenetic trees. *Rickettsia raoultii* infections in humans have been occasionally reported in China. In 2018, 26 human cases collected in three Medical Centers from Henan, Shandong, and Inner Mongolia were determined infected with *R. raoultii* [23]. Most of the patients only showed common nonspecific manifestations, such as fever and malaise. Our result revealed the genetic diversity of *R. raoultii* in this area. Their virulence and infectivity still need further exploration.

Three *Anaplasma* species were identified in this study. Except for *A. bovis*, all of them were detected in *I. persulcatus* ticks, indicating their host specificity. Of those, *A. phagocytophilum* is considered the most important human-pathogenic *Anaplasma* species. Human cases infected by *A. phagocytophilum* have been reported in multiple provinces of China [2, 24, 25]. In this study, two genotypes of *A. phagocytophilum* were identified. Although their 16S sequences were 100% identical, they separated into two distinct clades in the phylogenetic trees based on *gltA* and *groEL* sequences. This result may suggest the long-term evolution and recombination of *A. phagocytophilum* in this area. Notably, the type I strain was detected in an *I. persulcatus* tick from a goat, while the type II strains were all detected in *I. persulcatus* ticks from cattle. It is of interest whether there are any relationships between the animal hosts and the genetic types of *A. phagocytophilum*. In addition, a previously uncharacterized *Anaplasma* species closely related to *A. ovis* was detected. Herein we name it “*Candidatus* Anaplasma mongolica”.

*Ehrlichia muris* is an agent of human ehrlichiosis. In 2009, an *E. muris*–like agent was identified as a causative agent of human ehrlichiosis in the United States [26]. Although there have been several reports of *E. muris* in ticks from northeast China [27], only one single sequence was available in the GenBank Database to date. Our result may provide some information on the distribution and genetic characteristics of *E. muris* in China. Although no human cases infected by *E. muris* have been reported in China, our data suggest the potential risk of ehrlichiosis in this area. Out of our expectation, *Candidatu*s Lariskella sp. belonging to the family *Candidatus* Midichloriaceae, the order Rickettsiales, was detected. In 2004, *Candidatus* Lariskella arthropodarum has been detected in acutely febrile patients who have been bitten by *Ixodes* ticks in the Far East of Russia, suggesting that it may be a potential tick-borne human pathogen [28]. In 2022, we detected *Candidatu*s Lariskella sp. in *Ixodes* ticks from Guizhou Province, Southwest China, and obtained the *gltA* and *groEL* sequences of the genus *Ca.* Lariskella for the first time. We name it “*Candidatus* Lariskella guizhouensis” [9]. However, in this study, the 16S sequences of *Ca.* Lariskella strains we detected were 100% identical to *Ca.* Lariskella arthropodarum, while the *gltA* sequences were 100% identical to *Ca.* Lariskella guizhouensis in the absence of those of *Ca.* Lariskella arthropodarum strains. Based on the current data, it is hard to determine whether the detected strains should be classified as *Ca.* Lariskella arthropodarum or *Ca.* Lariskella guizhouensis. Actually, it is also quite possible that these two species be the same species.

*Borrelia miyamotoi* is an etiologic agent of relapsing fever. In 2021, an investigation performed in the same area reported *B. miyamotoi* infections in both ticks and humans [16]. Our result confirmed the circulation of *B. miyamotoi* in this area. In addition, *B. afzelii* and *B. garinii* were also detected. *Borreliella garinii* is the agent of Lyme disease widely distributed in China. To date, it has been reported in Heilongjiang, Jilin, Liaoning, Hebei, Inner Mongolia, Gansu, Zhejiang, and Xinjiang provinces in China, mainly located in north China [29, 30]. In this study, a high prevalence (8.08%) of *B. garinii* was observed in *I. persulcatus* ticks. Additionally, the detected strains showed considerable genetic polymorphism. Although Lyme disease is rarely reported in this area, our results suggest that local people may be at risk of Lyme disease infection.

To be noticed, environmental factors may affect the distribution of tick species, thus affecting the diversity and abundance of tick-borne pathogens. In this study, all the ticks were collected in forest areas of the Greater Khingan Range, Northeast China. Accordingly, most of the ticks (*I. persulcatus* and *H. concinna*) collected in this study ecologically fit biogeographic zones covered by coniferous forests with strong seasonality in temperature. As previously reported, *I. persulcatus* and *H. concinna* harbor an extremely high variety of tick-borne agents [1]. In this study, the remarkable diversity and abundance of tick-borne pathogens were identified, which is highly consistent with previous studies. This result also suggests that similar biogeographic zones may also warrant surveillance of tick-borne pathogens.

There are some limitations in this study. First, all the ticks were collected from a few domestic animals in one site, and the sample size was also small. Therefore, the tick species and the pathogens they carried may not be representative of this area. Second, because all the ticks were removed from domestic animals, it is possible that the detected pathogens are from the blood meal of ticks instead of the ticks themselves. In further study, testing tick-borne pathogens in host-seeking ticks and domestic animals may provide more useful information.

## Conclusion

In conclusion, 11 tick-borne bacterial pathogens were identified in Yakeshi City, Inner Mongolia. Some of them showed a high prevalence (*Ca*. Rickettsia tarasevichiae) and diverse genotypes (*R. raoultii* and *A. phagocytophilum*). These data suggest that Yakeshi City might be a potential hotspot of tick-borne diseases.

### Supplementary Information

**Additional file 1: Figure S1.** Phylogenetic trees based on the nucleotide sequences of 23S rRNA gene of *Candidatus *Anaplasma mongolica.

**Additional file 2: Table S1. **Genbank numbers of *Rickettsia*, *Anaplasma*, *Ehrlichia*, *Borrelia*, *Borreliella*, and *Candidatus* Lariskella sequences recovered in this study.

## Data Availability

All sequence files are available from the GenBank database (OR226549-OR226557, OR226561-OR226566, OR228866-OR228882, OR237106-OR237117, OR284777-OR284814, OR287185-OR287190, OR339536-OR339545) (Details shown in Table S1).
