# Supplementary material for: Molecular surveillance reveals a potential hotspot of tick-borne disease in Yakeshi City, Inner Mongolia
Source: BMC Microbiol. 2023 Nov 20;23:359. doi: 10.1186/s12866-023-03110-6 (PMC10662550; doi:10.1186/s12866-023-03110-6)
Supplement: Supplementary file 1 — Additional file 1: Figure S1. Phylogenetic trees based on the nucleotide sequences of 23S rRNA gene of Candidatus Anaplasma mongolica. [file 12866_2023_3110_MOESM1_ESM.docx]

**Figure S1.** Phylogenetic trees based on the nucleotide sequences of 23S rRNA gene of *Candidatus* Anaplasma mongolica.
