# Supplementary material for: Molecular surveillance reveals a potential hotspot of tick-borne disease in Yakeshi City, Inner Mongolia
Source: BMC Microbiol. 2023 Nov 20;23:359. doi: 10.1186/s12866-023-03110-6 (PMC10662550; doi:10.1186/s12866-023-03110-6)
Supplement: Supplementary file 2 — Additional file 2: Table S1. Genbank numbers of Rickettsia, Anaplasma, Ehrlichia, Borrelia, Borreliella, and Candidatus Lariskella sequences recovered in this study. [file 12866_2023_3110_MOESM2_ESM.docx]

Table S1. Genbank numbers of *Rickettsia*, *Anaplasma*, *Ehrlichia*, *Borrelia*, *Borreliella*, and *Candidatus* Lariskella sequences recovered in this study.

| No. | **Gene** | **Genbank number** | **Bacterial strain** |
| --- | --- | --- | --- |
| 1 | 16S | OR226549 | Borrelia_miyamotoi_N8 |
| 2 | 16S | OR226550 | Borrelia_miyamotoi_N16 |
| 3 | 16S | OR226551 | Borrelia_miyamotoi_N34 |
| 4 | 16S | OR226552 | Borreliella_garinii_N11 |
| 5 | 16S | OR226553 | Borreliella_garinii_N22 |
| 6 | 16S | OR226554 | Borreliella_garinii_N74 |
| 7 | 16S | OR226555 | Borreliella_garinii_N130 |
| 8 | 16S | OR226556 | Borreliella_garinii_N141 |
| 9 | 16S | OR226557 | Borreliella_afzelii_N136 |
| 10 | 16S | OR226561 | Ehrlichia_muris_N32 |
| 11 | 16S | OR226562 | Ehrlichia_muris_N37 |
| 12 | 16S | OR226563 | Ehrlichia_muris_N46 |
| 13 | 16S | OR226564 | Ehrlichia_muris_N53 |
| 14 | 16S | OR226565 | Ehrlichia_muris_N61 |
| 15 | 16S | OR226566 | Ehrlichia_muris_N142 |
| 16 | 16S | OR228871 | Candidatus_Rickettsia_tarasevichiae_N2 |
| 17 | 16S | OR228872 | Candidatus_Rickettsia_tarasevichiae_N18 |
| 18 | 16S | OR228873 | Candidatus_Rickettsia_tarasevichiae_N86 |
| 19 | 16S | OR228874 | Rickettsia_raoultii_N78_typeI |
| 20 | 16S | OR228875 | Rickettsia_raoultii_N83_typeI |
| 21 | 16S | OR228876 | Rickettsia_raoultii_N95_typeI |
| 22 | 16S | OR228877 | Rickettsia_raoultii_N84_typeII |
| 23 | 16S | OR228878 | Rickettsia_raoultii_N87_typeII |
| 24 | 16S | OR228879 | Rickettsia_raoultii_N98_typeII |
| 25 | 16S | OR228880 | Rickettsia_heilongjiangensis_N111 |
| 26 | 16S | OR228881 | Rickettsia_heilongjiangensis_N114 |
| 27 | 16S | OR228882 | Rickettsia_heilongjiangensis_N152 |
| 28 | 16S | OR228866 | Candidatus_Lariskella_sp._N5 |
| 29 | 16S | OR228867 | Candidatus_Lariskella_sp._N11 |
| 30 | 16S | OR228868 | Candidatus_Lariskella_sp._N21 |
| 31 | 16S | OR228869 | Candidatus_Lariskella_sp._N45 |
| 32 | 16S | OR228870 | Candidatus_Lariskella_sp._N143 |
| 33 | *gltA* | OR237106 | Candidatus_Rickettsia_tarasevichiae_N2 |
| 34 | *gltA* | OR237107 | Candidatus_Rickettsia_tarasevichiae_N18 |
| 35 | *gltA* | OR237108 | Candidatus_Rickettsia_tarasevichiae_N86 |
| 36 | *gltA* | OR237109 | Rickettsia_raoultii_N78_typeI |
| 37 | *gltA* | OR237110 | Rickettsia_raoultii_N83_typeI |
| 38 | *gltA* | OR237111 | Rickettsia_raoultii_N95_typeI |
| 39 | *gltA* | OR237112 | Rickettsia_raoultii_N84_typeII |
| 40 | *gltA* | OR237113 | Rickettsia_raoultii_N87_typeII |
| 41 | *gltA* | OR237114 | Rickettsia_raoultii_N98_typeII |
| 42 | *gltA* | OR237115 | Rickettsia_heilongjiangensis_N111 |
| 43 | *gltA* | OR237116 | Rickettsia_heilongjiangensis_N114 |
| 44 | *gltA* | OR237117 | Rickettsia_heilongjiangensis_N152 |
| 45 | *groEL* | OR284777 | Candidatus_Rickettsia_tarasevichiae_N2 |
| 46 | *groEL* | OR284778 | Candidatus_Rickettsia_tarasevichiae_N18 |
| 47 | *groEL* | OR284779 | Candidatus_Rickettsia_tarasevichiae_N86 |
| 48 | *groEL* | OR284780 | Rickettsia_raoultii_N78_typeI |
| 49 | *groEL* | OR284781 | Rickettsia_raoultii_N83_typeI |
| 50 | *groEL* | OR284782 | Rickettsia_raoultii_N95_typeI |
| 51 | *groEL* | OR284783 | Rickettsia_raoultii_N84_typeII |
| 52 | *groEL* | OR284784 | Rickettsia_raoultii_N87_typeII |
| 53 | *groEL* | OR284785 | Rickettsia_raoultii_N98_typeII |
| 54 | *groEL* | OR284786 | Rickettsia_heilongjiangensis_N111 |
| 55 | *groEL* | OR284787 | Rickettsia_heilongjiangensis_N114 |
| 56 | *groEL* | OR284788 | Rickettsia_heilongjiangensis_N152 |
| 57 | *gltA* | OR284789 | Ehrlichia_muris_N32 |
| 58 | *gltA* | OR284790 | Ehrlichia_muris_N37 |
| 59 | *gltA* | OR284791 | Ehrlichia_muris_N46 |
| 60 | *gltA* | OR284792 | Ehrlichia_muris_N53 |
| 61 | *gltA* | OR284793 | Ehrlichia_muris_N61 |
| 62 | *gltA* | OR284794 | Ehrlichia_muris_N142 |
| 63 | *groEL* | OR284795 | Ehrlichia_muris_N32 |
| 64 | *groEL* | OR284796 | Ehrlichia_muris_N37 |
| 65 | *groEL* | OR284797 | Ehrlichia_muris_N46 |
| 66 | *groEL* | OR284798 | Ehrlichia_muris_N53 |
| 67 | *groEL* | OR284799 | Ehrlichia_muris_N61 |
| 68 | *groEL* | OR284800 | Ehrlichia_muris_N142 |
| 69 | *gltA* | OR284801 | Candidatus_Lariskella_sp._N5 |
| 70 | *gltA* | OR284802 | Candidatus_Lariskella_sp._N11 |
| 71 | *gltA* | OR284803 | Candidatus_Lariskella_sp._N21 |
| 72 | *gltA* | OR284804 | Candidatus_Lariskella_sp._N45 |
| 73 | *gltA* | OR284805 | Candidatus_Lariskella_sp._N143 |
| 74 | *flaB* | OR284806 | Borrelia_miyamotoi_N8 |
| 75 | *flaB* | OR284807 | Borrelia_miyamotoi_N16 |
| 76 | *flaB* | OR284808 | Borrelia_miyamotoi_N34 |
| 77 | *flaB* | OR284809 | Borreliella_garinii_N11 |
| 78 | *flaB* | OR284810 | Borreliella_garinii_N22 |
| 79 | *flaB* | OR284811 | Borreliella_garinii_N74 |
| 80 | *flaB* | OR284812 | Borreliella_garinii_N130 |
| 81 | *flaB* | OR284813 | Borreliella_garinii_N141 |
| 82 | *flaB* | OR284814 | Borreliella_afzelii_N136 |
| 83 | 16S | OR287185 | Anaplasma_phagocytophilum_N3 |
| 84 | 16S | OR287186 | Anaplasma_phagocytophilum_N54 |
| 85 | 16S | OR287187 | Anaplasma_phagocytophilum_N55 |
| 86 | 16S | OR287188 | Anaplasma_phagocytophilum_N136 |
| 87 | 16S | OR287189 | Anaplasma_bovis_N115 |
| 88 | 16S | OR287190 | Anaplasma_sp._N127 |
| 89 | *gltA* | OR339536 | Anaplasma_phagocytophilum_N3 |
| 90 | *gltA* | OR339537 | Anaplasma_phagocytophilum_N54 |
| 91 | *gltA* | OR339538 | Anaplasma_phagocytophilum_N55 |
| 92 | *gltA* | OR339539 | Anaplasma_phagocytophilum_N136 |
| 93 | *gltA* | OR339540 | Anaplasma_sp._N127 |
| 94 | *groEL* | OR339541 | Anaplasma_phagocytophilum_N3 |
| 95 | *groEL* | OR339542 | Anaplasma_phagocytophilum_N54 |
| 96 | *groEL* | OR339543 | Anaplasma_phagocytophilum_N55 |
| 97 | *groEL* | OR339544 | Anaplasma_bovis_N115 |
| 98 | *groEL* | OR339545 | Anaplasma_sp._N127 |
